# mTORC1 phosphorylates LARP6 to stimulate type I collagen expression

**DOI:** 10.1038/srep41173

**Published:** 2017-01-23

**Authors:** Yujie Zhang, Branko Stefanovic

**Affiliations:** 1 Department of Biomedical Sciences, College of Medicine, Florida State University, Tallahassee, Florida 32306, USA

## Abstract

Excessive deposition of type I collagen causes fibrotic diseases. Binding of La ribonucleoprotein domain family, member 6 (LARP6) to collagen mRNAs regulates their translation and is necessary for high type I collagen expression. Here we show that mTORC1 phosphorylates LARP6 on S348 and S409. The S348A/S409A mutant of LARP6 acts as a dominant negative protein in collagen biosynthesis, which retards secretion of type I collagen and causes excessive posttranslational modifications. Similar effects are seen using mTORC1 inhibitor rapamycin or by knocking down raptor. The S348A/S409A mutant weakly interacts with the accessory protein STRAP, needed for coordinated translation of collagen mRNAs. The interaction of wt LARP6 and STRAP is also attenuated by rapamycin and by raptor knockdown. Additionally, in the absence of S348/S409 phosphorylation LARP6 is sequestered in increasing amounts at the ER membrane. We postulate that phosphorylation of S348/S409 by mTORC1 stimulates the interaction of LARP6 and STRAP to coordinate translation of collagen mRNAs and to release LARP6 from the ER for new round of translation. These mechanisms contribute to high level of collagen expression in fibrosis.

Type I collagen is the most abundant protein in the human body. It is composed of two α1(I) and one α2(I) polypeptides which fold into triple helix^1^. Type I collagen is expressed at high levels in bone, skin, tendons and connective tissue^2^. In fibrosis, excessive synthesis of collagen occurs in parenchymal organs, leading to scarring and loss of function^3^. To understand normal tissue development, as well as pathogenesis of fibrosis, it is important to elucidate molecular mechanisms regulating collagen expression. Compelling evidence has shown that collagen expression is primarily regulated at the posttranscriptional level, including regulation of half-life and translation of collagen mRNAs^4^,^5^,^6^,^7^. Binding of RNA binding protein La ribonucleoprotein domain family, member 6 (LARP6) to the conserved structural element in the 5′UTR of collagen α1(I) and α2(I) mRNAs (5′ stem-loop) (5′SL) regulates their translation^8^,^9^,^10^,^11^. LARP6 tethers collagen mRNAs to the cytoskeletal filaments; nonmuscle myosin and vimentin^9^,^12^. The association with myosin is necessary for partitioning of collagen mRNAs to the ER membrane^8^. LARP6 also recruits two accessory factors for translation initiation; RNA helicase A (RHA) and serine-threonine kinase receptor-associated protein (STRAP)^13^,^14^. These factors coordinate translation of collagen mRNAs so that synthesis of collagen α1(I) is coupled to that of α2(I). This allows efficient folding of the polypeptides into heterotrimer. Association with vimentin filaments prolongs the half-life of collagen mRNAs, further contributing to the high level of synthesis. So, comprehensive understanding of the LARP6-dependent mechanism of type I collagen synthesis is needed to provide new therapeutic targets for fibrosis.

mTOR (mammalian target of rapamycin) is a serine/threonine kinase that is assembled into two different multiprotein complexes, mTOR complex 1 (mTORC1) and 2 (mTORC2)^15^,^16^,^17^,^18^,^19^. mTORC2 is involved in actin polymerization, cell spreading, activation of the kinase AKT by phosphorylation on S473 and regulation of its downstream biological functions^18^,^20^,^21^, while mTORC1 is activated by a variety of stimuli, including growth factors, insulin, or amino acids, to regulate translation through phosphorylation of two downstream effectors, translational factor 4E binding protein 1 (4E-BP1) and p70 ribosomal S6 kinase (S6K)^22^,^23^,^24^. Thus, activation of mTOR pathway results in stimulation of translation, reorganization of cytoskeletal filaments, cell growth, survival and proliferation.

Rapamycin, an inhibitor of mTORC1, was initially introduced as an immunosuppressive drug^25^,^26^. We and others have shown that rapamycin has anti-fibrotic effect in animal models of hepatic, renal, and pulmonary fibrosis^27^,^28^,^29^,^30^ and we have suggested that the underlying anti-fibrotic mechanism of rapamycin may involve alteration of LARP6 function. Recently, we reported that LARP6 is phosphorylated at eight serines, but that phosphorylation of S451 by AKT is necessary for other phosphorylations to take place and for activation of LARP6 in collagen biosynthesis^31^. Five of these other phosphorylation sites conform to the mTOR consensus sequence, so this study was performed to establish whether mTOR participates in activation of LARP6. Here, we report that mTORC1 phosphorylates LARP6 at S348/S409 and that lack of these phosphorylations has a dominant negative effect on type I collagen biosynthesis. We also provide evidence that mTORC1-dependent phosphorylation of LARP6 is required for recruitment of STRAP and for proper subcellular trafficking of LARP6.

## Results

### Inhibitors of mTOR pathway alter phosphorylation of LARP6

**Figure 1:**
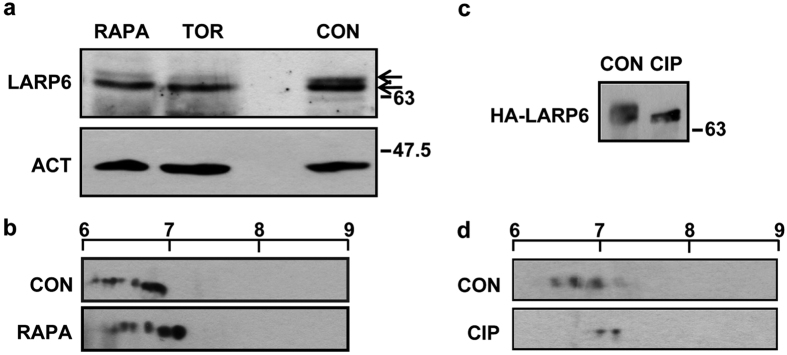
mTOR inhibitors alter LARP6 phosphorylation. (**a**) Reduced phosphorylation of endogenous LARP6. HLFs were treated with DMSO (CON), rapamycin (RAPA) and Torin 1 (TOR), and analyzed by Western blot. Arrows: phosphorylated isoforms of LARP6. Loading control: β-actin (ACT). (**b**) Rapamycin changes pI of LARP6. Cell lysates of HLFs treated with CON or RAPA were subjected to 2DGE followed by Western blot. The scale at the top indicates the pH. (**c**) Removal of phosphates from LARP6 by calf intestinal phosphatase (CIP). HA-LARP6 was immunoprecipitated and digested without (CON) or with CIP and analyzed by Western blot. (**d**) Change in pI of LARP6 by CIP. The samples from (**c**) were analyzed by 2DGE and Western blot.

We have reported that LARP6 is phosphorylated at eight serines and that AKT is required for S451 phosphorylation^31^. For full understanding of the role of LARP6 in regulating collagen expression it was important to characterize the other phosphorylation sites. Among the eight sites, five resemble mTOR consensus sequence, which prefers a proline, a hydrophobic or an aromatic residue at the +1 position^32^. To assess if these sites are mTOR targets, human lung fibroblasts (HLFs) were treated with mTORC1 and mTORC1/2 inhibitors, rapamycin and Torin 1^33^. In one dimensional SDS-PAGE (1DGE), endogenous LARP6 migrated as two bands (Fig. 1a, arrows), representing differently phosphorylated LARP6 isoforms^10^. The slower migrating band was markedly reduced by mTOR inhibitors (Fig. 1a, lanes 1 and 2), indicating that some phosphorylations of LARP6 may be mTOR-dependent. To verify this, we employed 2DGE, where endogenous LARP6 was resolved as series of isoforms with isoelectric point (pI) ranging from 6.0–7.0. However, upon rapamycin treatment LARP6 showed a pI shift towards the more basic region, from 6.2–7.2 (Fig. 1b).

To verify that the signals on 1DGE and 2DGE represent LARP6 molecules carrying different phosphorylations, we treated the immunoprecipitated HA-LARP6 with calf intestinal alkaline phosphatase (CIP). In 1DGE HA-LARP6 was resolved as two bands and the upper band disappeared with CIP treatment (Fig. 1c). In 2DGE, LARP6 was resolved as multiple molecular species with pI ranging from 6.4–7.2. After CIP treatment only two molecular species remained, with pI of 7.0 and 7.2 (Fig. 1d). The drastic pI change by CIP suggests that most signals resolved on 2DGE represent differentially phosphorylated LARP6. We concluded that mTOR inhibitors can alter the phosphorylation status of LARP6.

### Inhibitor of mTORC1, rapamycin, decreases secretion of type I collagen into cellular medium

**Figure 2:**
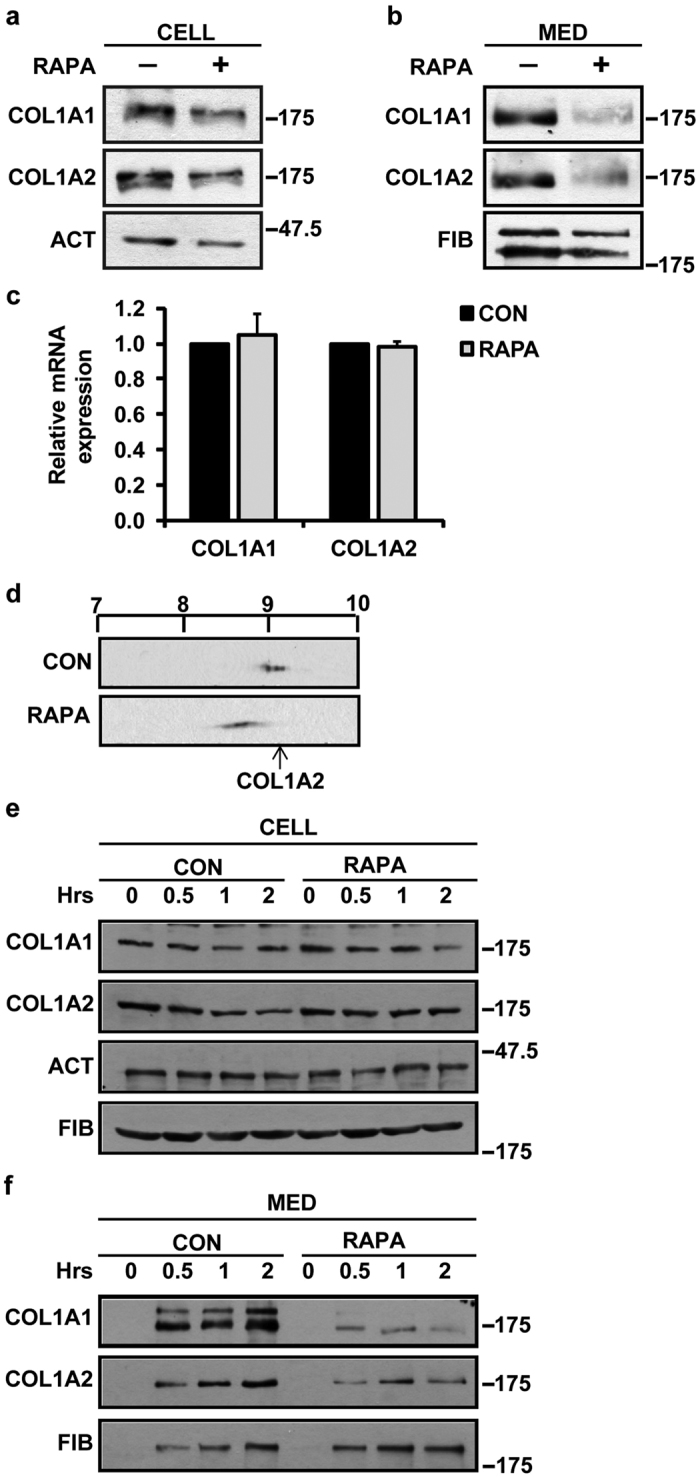
Inhibition of mTORC1 reduces secretion of type I collagen. (**a**) Intracellular collagen polypeptides in HLFs treated with RAPA. HLFs were treated with CON or RAPA for 24 h and intracellular (CELL) level of collagen α1(I) (COL1A1) and α2(I) (COL1A2) polypeptides was analyzed by Western blot. Loading control: β-actin (ACT). (**b**) Level of collagen polypeptides in the cellular medium. Collagen polypeptides in the medium (MED) of cells in (**a**) were analyzed by Western blot. Loading control: fibronectin (FIB). (**c**) Rapamycin does not change expression of collagen mRNAs. Total RNA from cells in (**a**) was analyzed for expression of COL1A1 and COL1A2 mRNA by real-time RT-PCR. Expression was normalized to the internal control β-actin mRNA and presented as mean ± SD (n = 3). (**d**) Change in pI of COL1A2 by rapamycin. COL1A2 from control HLFs or HLFs treated with RAPA for 24 h was analyzed by 2DGE. Arrow: isoelectric focusing of COL1A2 in control cells. (**e**) Time course of inhibition of type I collagen by rapamycin. HLFs were treated with RAPA for the indicated time periods and intracellular (CELL) level of collagen polypeptides was analyzed by Western blot. CON; untreated cells. (**f**) Time course of inhibition of collagen secretion. Collagen in the medium of cells in (**e**) was analyzed by Western blot.

Having shown that mTOR is involved in phosphorylating LARP6, it was important to assess the effects of mTOR inhibition on collagen expression. The intracellular collagen was not affected by rapamycin (Fig. 2a), however, secretion of both collagen polypeptides was drastically reduced (Fig. 2b). Rapamycin did not change the expression of collagen α1(I) or α2(I) mRNAs (Fig. 2c), suggesting that the primary effect of mTORC1 inhibition is reduced collagen excretion.

Type I collagen is produced by folding of two α1(I) and one α2(I) polypeptides into a trimer after they are posttranslationally modified. Posttranslational modifications include hydroxylations of prolines/lysines and glycosylations^1^. Delayed folding of the triple helix results in excessive modification of collagen polypeptides, primarily due to excessive hydroxylations of prolines and lysines^1^,^34^,^35^. Although hydroxylations do not contribute to the charge of a protein, hydroxylations of lysines partially neutralize positive charge, resulting in more acidic pI of the polypeptides. This pI shift has been observed in patients with type I collagen folding defect^36^,^37^, therefore, it can be used as a readout of perturbed collagen synthesis. To assess if reduced secretion of collagen is associated with its hyper-modifications we analyzed the pI of collagen α2(I) polypeptide. The α1(I) polypeptide was not amenable to analysis, because the antibody could not recognize this polypeptide after isoelectric focusing. In control cells α2(I) polypeptide had the pI around 9.0 (arrow, Fig. 2d), but the pI was shifted to more acidic region by rapamycin, suggesting that hyper-modified polypeptides had been produced.

In the above experiments rapamycin treatment was 24 h, it was necessary to show if similar effects can be achieved with shorter time, when kinase inhibitors usually show an effect. While rapamycin did not alter intracellular collagen at 0.5, 1, and 2 h (Fig. 2e), the accumulation of collagen polypeptides into the cellular medium was lower at 0.5 h for the treated cells and did not increase further (Fig. 2f, RAPA). Control cells showed higher accumulation after 0.5 h, with further increase by 2 h (Fig. 2f, CON). These results suggest that secretion of collagen is compromised 30 min after mTORC1 inhibition.

### Serines 348/409 of LARP6 are targets of mTORC1 phosphorylation

**Figure 3:**
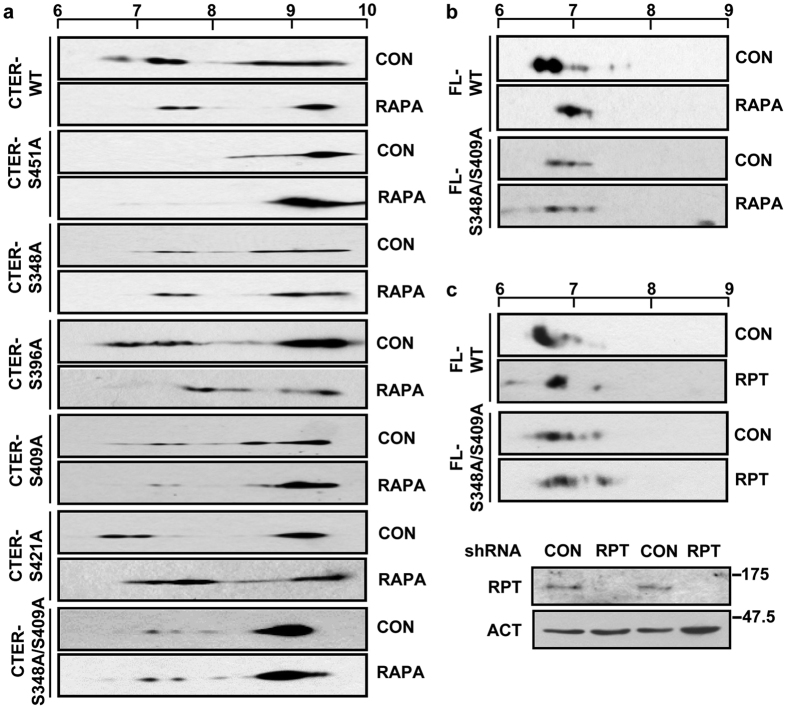
Identification of mTORC1 dependent phosphorylation sites. (**a**) Phosphorylations of the C-terminal domain (CTER) of LARP6. Wt CTER (panels 1 and 2) and CTER carrying single alanine mutations (S451A, S348A, S396A, S409A, S421A; panels 3–12) or a double alanine mutant, S348A/S409A (panels 13 and 14), were expressed in HEK293 cells, treated with rapamycin (RAPA) or untreated (CON) and analyzed by 2DGE and Western blot. (**b**) Effect of rapamycin of the pI of full size LARP6. Wt HA-LARP6 and S348A/S409A mutant of the full size LARP6 were analyzed by 2DGE. (**c**) pI change of full size LARP6 in raptor knockdown cells. Raptor was knocked down in HLFs by raptor specific shRNA (RPT) and the pI wt HA-LARP6 and S348A/S409A mutant was analyzed by 2DGE. CON, scrambled shRNA. Lower panels: expression of raptor (RPT) in two knockdown and control samples.

All serines which are candidates for mTOR phosphorylation are within the C-terminus of LARP6 (CTER). To identify which serines are mTOR targets we individually mutated S348, S396, S409, S421 and S451 into alanines and expressed the CTER mutants in the presence or absence of rapamycin. We surmised that if a serine is mTOR target, its mutation into alanine will abolish rapamycin-induced pI alteration. Wt CTER showed a broad range of pI (Fig. 3a, panel 1) and several signals were abolished by rapamycin (Fig. 3a, panel 2), suggesting that some phosphorylations are rapamycin sensitive. Phosphorylation of S451 is a prerequisite for other phosphorylations^31^, so this mutant does not show the multiple phosphorylated isoforms and does not respond to rapamycin (panels 3 and 4). When S348A, S396A, S409A, and S421A were analyzed, they all showed a pI alteration by rapamycin (Fig. 3a, panels 5–12), indicating that they are still rapamycin sensitive and that, perhaps, multiple serines need to be mutated to observe the effect. We found a double mutant S348A/S409A was resistant to rapamycin, because its pI did not change with rapamycin (Fig. 3a, panels 13 and 14). This suggests that mTORC1-dependent phosphorylation cannot be observed if S348/S409 are changed into alanines.

We also generated S348A/409A in the FL HA-LARP6 and tested its response to rapamycin. As shown in Fig. 3b, a similar result was observed for FL HA-LARP6 as CTER. This corroborated the finding that S348/S409 are the targets of mTOR.

To provide further evidence that mTORC1 signaling pathway targets LARP6 on S348/S409, we knocked down the regulatory protein of mTORC1, raptor^15^,^38^,^39^. Raptor knockdown reduced phosphorylation of wt HA-LARP6, as evidenced by pI shift into more basic region (Fig. 3c, compare panels 1 and 2). This pI shift was similar to that seen with rapamycin (Fig. 3b, panels 1 and 2). However, raptor knockdown did not change the pI of S348A/S409A (Fig. 3c, panels 3 and 4), strongly suggesting that S348/S409 phosphorylations are mTORC1-dependent.

### LARP6 phosphorylation changes in activation of collagen-nonproducing cells into collagen-producing cells

HLFs produce high level of collagen constitutively and LARP6 phosphorylation in these cells reflects its status in terminally differentiated fibroblasts. To provide insight if LARP6 phosphorylation is a dynamic process in differentiation of collagen-nonproducing cells into collagen-producing cells we used culture activation of hepatic stellate cells (HSCs). HSCs are cells which produce type I collagen in liver fibrosis^40^. Freshly isolated HSCs from normal rat liver are in quiescent state and synthesize trace amount of collagen. However, HSCs spontaneously activate into myofibroblasts by culturing *in vitro* and increase collagen expression 50–100 fold^40^,^41^,^42^, mimicking the changes which HSC undergo in liver fibrosis. The activation starts after 3 days in culture and is well advanced by day 5. Therefore, we analyzed HSCs after 3 days and after 5 days in culture to investigate whether there is a change in LARP6 phosphorylation.

**Figure 4:**
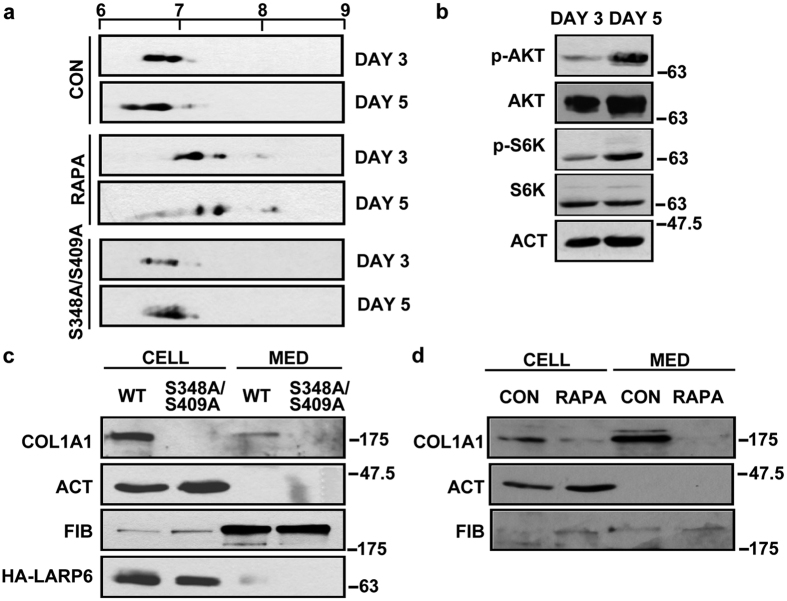
Dynamic changes in phosphorylation of LARP6 in HSCs. (**a**) LARP6 phosphorylation changes in activation of HSCs. Primary rat HSCs were transduced by adenovirus expressing wt LARP6 on day 2 or on day 4 and analyzed by 2DGE on day 3 or on day 5 (CON, panels 1 and 2). The same cells were also treated with rapamycin on day 2 and day 4 and analyzed on day 3 and day 5 (panels 3 and 4). Panels 5 and 6; cells transduced with S348A/S409A mutant. (**b**) Increased AKT and mTORC1 signaling during HSCs activation. HSCs at day 3 and day 5 in culture were analyzed for expression of phospho-AKT and phospho-S6K. p-AKT: phopho-AKT. AKT: total AKT. p-S6K: phospho-S6K. S6K: total S6K. ACT: β-actin, loading control. (**c**) Dominant negative effect of S348A/S409A in HSCs. HSCs were transduced on day 3 with wt LARP6 and S348A/S409A mutant and on day 5 cellular (CELL) and medium (MED) level of COL1A1 was analyzed by Western blot. Loading controls: β-actin (ACT) and fibronectin (FIB). HA-LARP6, expression of the transduced proteins. (**d**) Rapamycin reduces expression of collagen by HSCs. HSCs cultured for 5 days were treated with or without RAPA for 2 h and COL1A1 polypeptide was analyzed intracellularly (CELL) or in the medium (MED).

In day 3 HSCs, LARP6 had the pI from 6.5–7.0 (Fig. 4a, panel 1), but by day 5 the pI shifted toward the more acidic range of 6.1–6.8 (Fig. 4a, panel 2). This alteration suggested that LARP6 undergoes additional phosphorylations in transition of HSCs from quiescent into activated state.

When rapamycin treated HSCs were analyzed at day 3, LARP6 exhibited more basic pI (6.8–7.9) (Fig. 4a, compare panel 1 and 3). This indicates that some phosphorylations of LARP6 are rapamycin sensitive even in quiescent HSCs. However, when day 5 HSCs were analyzed, a more dramatic pI shift with rapamycin was observed; from 6.1–6.8 to 6.8–7.9 (Fig. 4a, compare panels 2 and 4). This suggests that the additional phosphorylations observed in day 5 HSCs are also affected by inhibiting mTORC1.

Mapping of the mTORC1-dependent phosphorylation sites to S348/S409 allowed us to test if this mutant can undergo the dynamic phosphorylation changes during HSCs activation. The pI of S348A/S409A did not reveal an increase in phosphorylation at day 5 (Fig. 4a, panels 5 and 6). This suggests that mTORC1-dependent S348/S409 phosphorylation is responsible for the increased phosphorylation of LARP6 during *in vitro* activation of HSCs. These indicate a correlation between S348/S409 phosphorylation and the ability to upregulate collagen.

To assess if there is an increased activity of AKT and mTORC1 signaling in HSCs between day 3 and day 5 in culture we analyzed expression of phosphorylated AKT and phosphorylated S6K. Increased phosphorylation of AKT and S6K was observed at day 5 compared to day 3, although the total AKT and S6K expression was comparable (Fig. 4b). These results indicate that there is an activation of AKT and mTOR signaling during HSCs activation, correlating with increased LARP6 phosphorylation.

### Dominant negative effect of S348A/S409A on type I collagen expression

The experiments in HSCs suggested that mTOR-dependent phosphorylation of LARP6 correlates with activation of collagen expression. To assess the functional significance of LARP6 phosphorylation we overexpressed S348A/S409A in HSCs and assessed its effect on collagen expression. We could not analyze α2(I) polypeptide, because our antibody could not recognize the rodent polypeptide. At day 5, HSCs expressing wt HA-LARP6 showed high expression of α1(I) polypeptide intracellularly and in the medium (Fig. 4c, top panel, lanes 1 and 3). The cellular level and the secretion of α1(I) polypeptide were undetectable in HSCs overexpressing S348A/S409A (lanes 2 and 4). Thus, lack of phosphorylaitons on S348/S409 in HSCs shuts down type I collagen synthesis.

Since S348/S409 phosphorylation is dependent on mTOR, we expected that rapamycin treatment of HSCs will have a similar effect as S348A/S409A. Rapamycin reduced both, the cellular level and the secretion of collagen into the cellular medium (Fig. 4d), suggesting a similar effect on collagen expression as the S348A/S409A.

**Figure 5:**
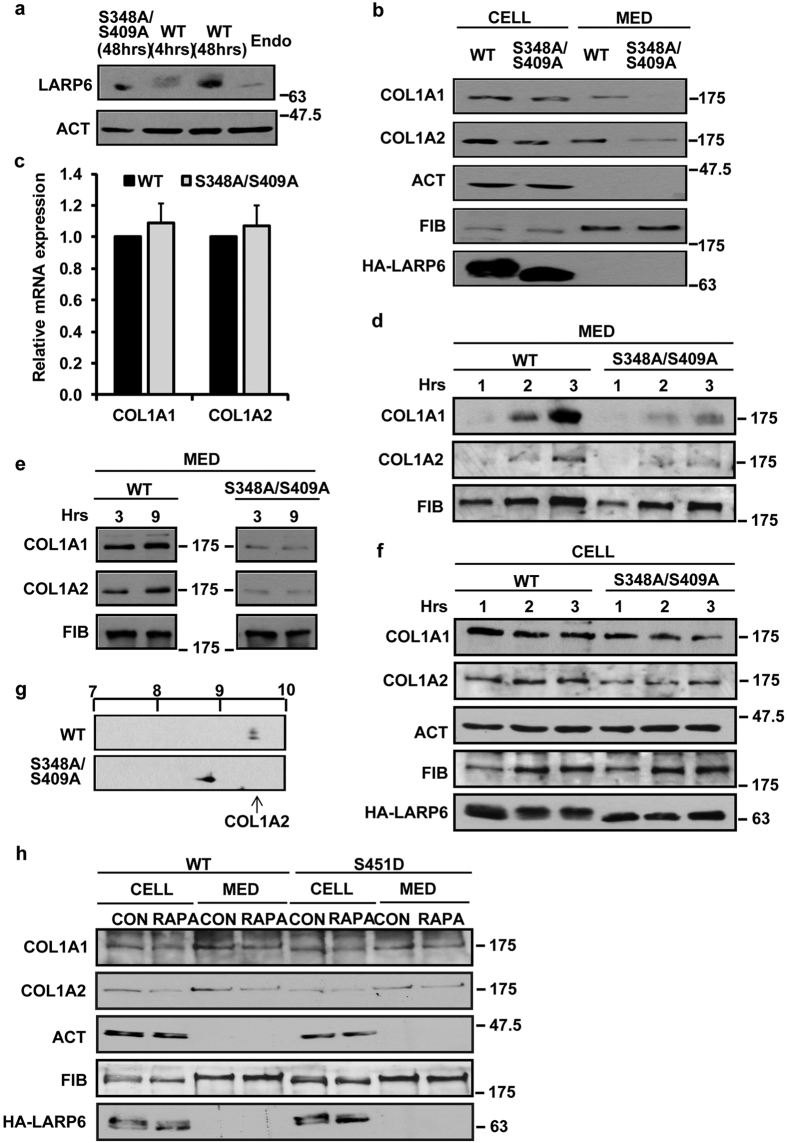
Dominant negative effect of S348A/S409A on collagen secretion from HLFs. (**a**) Expression level of transduced proteins. HLFs were transduced with adenovirus expressing S348A/S409A for 48 h (lane 1) or wt HA-LARP6 for 4 h (lane 2) or 48 h (lane 3) and analyzed by Western blot using anti-LARP6 antibody. Lane 4; expression of endogenous LARP6. β-actin (ACT): loading control. (**b**) Dominant negative effect in HLFs. HLFs were transduced with wt HA-LARP6 or S348A/S409A mutant and collagen polypeptides (COL1A1 and COL1A2) were analyzed intracellularly (CELL) and in the medium (MED). FIB: fibronectin loading control. (**c**) Expression of collagen mRNAs. Total RNA from cells in (**b**) was analyzed for expression of COL1A1 and COL1A2 mRNAs by real-time PCR. Relative expression normalized to β-actin mRNA is shown as mean ± SD (n = 3). (**d**) Secretion rate of collagen from HLFs expressing S348A/S409A. Wt HA-LARP6 and S348A/S409A mutant were expressed in HLFs and accumulation of collagen polypeptides in the medium was analyzed at indicated time points. Loading control: fibronectin (FIB). (**e**) Collagen polypeptides are not degraded in the cellular medium. Cellular medium from the 3 h time point in (**d**) was incubated for additional 6 h at 37 °C and analyzed by Western blot. (**f**) S348A/S409A mutant does not change intracellular level of collagen. Cells in (**d**) were analyzed for intracellular collagen level. (**g**) Hyper-modifications of COL1A2 polypeptide in cells expressing S348A/S409A. The pI of COL1A2 polypeptide in cells expressing wt LARP6 and S348A/S409A was analyzed by 2DGE. Arrow: pI of COL1A2 in control cells. (**h**) S451D mutant confers resistance to rapamycin. HLFs were transduced with wt HA-LARP6 or S451D mutant, treated with (RAPA) or without (CON) rapamycin and collagen polypeptides (COL1A1 and COL1A2) were analyzed intracellularly (CELL) and in the medium (MED). Fibronectin (FIB) and actin (ACT) were used as loading control. HA-LARP6, expression of transfected proteins.

To extend the finding that S348A/S409A is dominant negative for collagen production we repeated the experiment in HLFs. To obtain an idea how much S348A/S409A is overexpressed in the dominant negative experiments we compared the expression of endogenous LARP6 and transduced wt LARP6 and S348A/S409A. The dominant negative effect was analyzed 48 h after overexpression of S348A/S409A and at this time point its expression was about 10-fold higher than that of endogenous LARP6 (Fig. 5a). Wt HA-LARP6 expressions at 48 h and 4 h are also shown. The 4 h expression was comparable to the endogenous LARP6 and this time point was utilized for analyzing the subcellular localization of LARP6 shown later in the manuscript.

S348A/S409A overexpression in HLFs slightly reduced the intracellular levels of both polypeptides. However, polypeptides secretion was dramatically reduced (Fig. 5b, top panels). The steady-state levels of both collagen mRNAs were similar in HLFs expressing wt LARP6 and S348A/S409A (Fig. 5c). The reduced secretion of collagen polypeptides by S348A/S409A resembles the result obtained with rapamycin (Fig. 2). This suggests that phosphorylation of S348/S409 by mTORC1 regulates collagen production by HLFs.

### S348A/S409A decreases the rate of secretion of type I collagen

The decreased level of collagen found in the cellular medium by S348A/S409A or by rapamycin, suggests either the inefficient secretion of the protein or its accelerated degradation in the medium. To assess the secretion rate of collagen, we analyzed the accumulation of collagen α1(I) and α2(I) polypeptides in the medium after 1, 2, and 3 h. In cells overexpressing wt LARP6, a continuous increase in accumulation of collagen polypeptides in the cellular medium was observed within 3 h (Fig. 5d, lanes 1–3). However, their accumulation in the medium of cells overexpressing S348A/S409A was greatly retarded (Fig. 5d, lanes 4–6), suggesting that the mutant inhibited the rate of secretion of type I collagen.

To exclude the possibility that collagen polypeptides are subjected to more rapid degradation in cells overexpressing S348A/S409A, we collected the cellular medium at 3 h time point and incubated this medium for additional 6 h. The level of collagen polypeptides remained unchanged after the prolonged incubation (Fig. 5e), suggesting that medium did not contain proteolytic enzymes that would degrade collagen.

We also measured the intracellular level of collagen polypeptides and it remained constant (Fig. 5f), indicating that collagen peptides were made in the cell. The slow excretion of collagen from S348A/S409A expressing cells was not accompanied by the increased intracellular retention, probably because the excessive polypeptides were degraded inside the cells. These results suggest that lack of mTORC1-dependent LARP6 phosphorylation results in inefficient secretion of collagen.

Next, we assessed if the slow secretion of collagen polypeptides by S348A/S409A is associated with their hyper-modifications. Hyper-modifications are indicative of inefficient folding as the reason for slow secretion. The pI of collagen α2(I) polypeptide in control cells was around 9.5 (Fig. 5g, upper panel, arrow). S348A/S409A resulted in pI shift to 8.8, indicating hyper-modifications (Fig. 5g, lower panel). A similar shift was observed with rapamycin (Fig. 2d). Therefore, we concluded that inability to phosphorylate LARP6 by mTORC1 impairs proper folding of collagen, resulting in hyper-modifications of polypeptides and their inefficient secretion.

The S451D mutation of LARP6 results in constitutive phosphorylations at multiple other sites, so using this mutant provides an opportunity to study the effects of constitutively hyperphosphorylated LARP6 in cells. We have described this mutant in our previous work^31^. We surmised that the activity of S451D may be independent on mTOR, because the protein is already hyperphosphorylated. We overexpressed S451D and analyzed if it could overcome the inhibitory effect of rapamycin on collagen expression. We found that while rapamycin reduced secretion of collagen polypeptides from cells overexpressing WT LARP6, it did not alter intracellular collagen levels or the amount secreted into the medium of cells overexpressing S451D (Fig. 5h, lanes 3, 4, 7 and 8).

### Phosphorylation of S348/S409 is required for effective interaction with essential cofactor for translation of collagen mRNAs, STRAP

**Figure 6:**
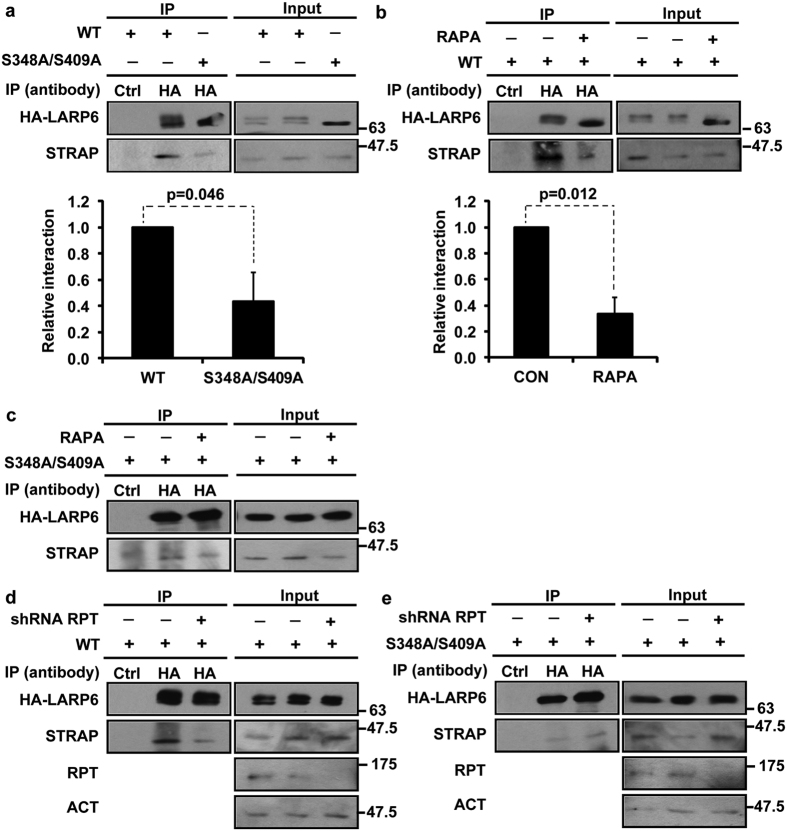
S348/S409 phosphorylation enhances the interaction of LARP6 and STRAP. (**a**) Interaction of S348A/S409A with STRAP. HLFs were transduced with wt HA-LARP6 and S348A/S409A mutant. Immunoprecipitation (IP) was done with control (lane 1) or anti-HA antibody (lanes 2 and 3) and the pulled down was analyzed using anti-HA (upper left panel) and anti-STRAP antibody (lower left panel). Right panels: expression of proteins in 10% of input. Graph: Relative interaction of LARP6 and STRAP calculated from three independent experiments. Signal of STRAP in the IP was normalized to the signal of LARP6 in the same IP and arbitrarily set as 1 for wt LARP6. Data are shown as mean ± SD. (**b**) Rapamycin decreases the interaction between LARP6 and STRAP. HLFs were transduced with wt HA-LARP6 and treated with (lane 3) or without (lanes 1 and 2) rapamycin (RAPA). IP was done as in (**a**). Graph: quantification of the relative interaction as in (**a**). (**c**) Interaction of S348A/S409A and STRAP is not affected by rapamycin. Experiment as in (**b**), except that S348A/S409A mutant was expressed. (**d**) Raptor knockdown reduces the LARP6/STRAP interaction. Raptor was knocked down in HLFs by raptor specific shRNA (RPT) (lane 3), while scrambled shRNA was used as control (lanes 1 and 2). Wt HA-LARP6 was then transduced and IP done as in (**a**). (**e**) Raptor knockdown has no effect on the S348A/S409A/STRAP interaction. Experiment was done as in (**d**), except S348A/S409A mutant was expressed.

To elucidate the underlying mechanism of poor secretion of collagen caused by S348A/S409A, we analyzed if this mutation affects the interaction of LARP6 with factors involved in collagen biosynthesis; STRAP and RHA^12^,^13^,^14^. STRAP is the cofactor which coordinates translation of collagen α1(I) and α2(I) mRNAs and in the absence of STRAP cells hyper-modify collagen polypeptides and inefficiently secrete collagen. We found that wt HA-LARP6 efficiently pulled down STRAP, while S348A/S409A showed less interaction with STRAP (Fig. 6a, left panel, compare lanes 2 and 3). The relative interaction of STRAP and LARP6 is shown at the bottom of Fig. 6a and indicates that about 50% less STRAP was pulled down with S348A/S409A.

We also analyzed if the interaction between LARP6 and STRAP is weaken by rapamycin or raptor knockdown. Rapamycin decreased the interaction between wt HA-LARP6 and STRAP (Fig. 6b, left panel, compare lanes 2 and 3); quantification of the interaction suggested that only 1/3 of STRAP was pulled down with wt HA-LARP6 after the rapamycin treatment (bottom panel, Fig. 6b). However, rapamycin did not change the already weak interaction of S348A/S409A with STRAP (Fig. 6c, left panel, compare lanes 2 and 3). Similar results were observed with raptor knockdown (Fig. 6d and e). Taken together, these results suggest that mTORC1-dependent S348/S409 phosphorylation is required for more efficient recruitment of STRAP.

### Phosphorylation of S348/S409 regulates subcellular distribution of LARP6

**Figure 7:**
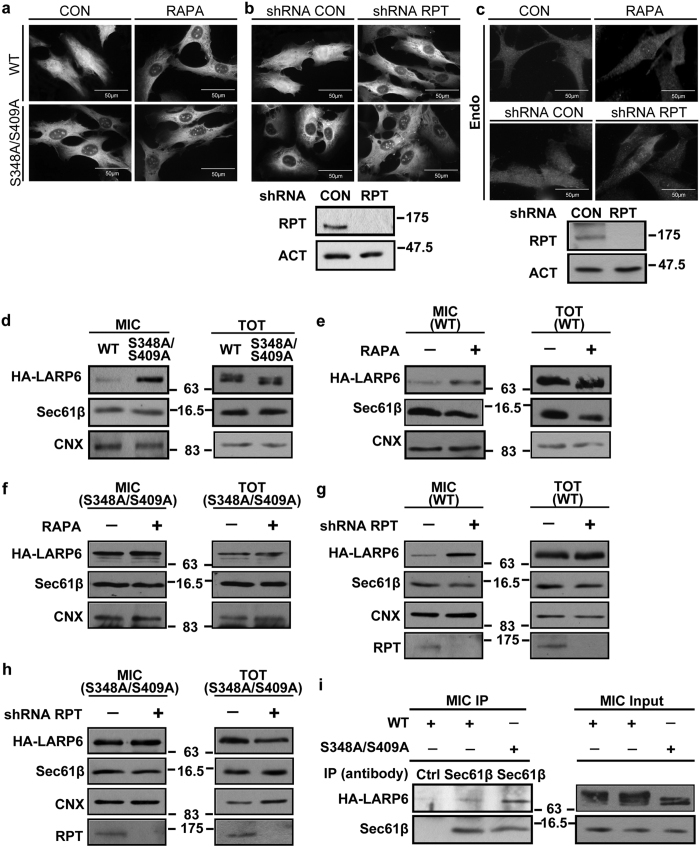
Association of LARP6 with ER membrane. (**a**) Cellular localization of HA-LARP6. Wt HA-LARP6 and S348A/S409A were expressed in HLFs for 4 h and immunostained in control (CON) and rapamycin (RAPA) treated cells using anti-HA antibody. (**b**) Localization of HA-LARP6 after raptor knockdown. Raptor was knocked down by shRNA (shRPT), wt HA-LARP6 and S348A/S409A were expressed and immunostained as in (**a**). Right panel: the efficiency of raptor knockdown (RPT), loading control: β-actin (ACT). (**c**) Cellular localization of endogenous LARP6. HLFs were treated without or with RAPA (upper panels) or transduced with lentivirus expressing control shRNA (CON) or shRNA raptor (RPT) (lower panels) and immunostained with anti-LARP6 antibody. Right panel: the efficiency of raptor knockdown (RPT). (**d**) Accumulation of S348A/S409A mutant in the microsomal fraction. Wt HA-LARP6 and S348A/S409A were expressed in HLFs and microsomal fraction (MIC, left panel) and total lysate (TOT, right panel) were analyzed by Western blot. Calnexin (CNX) and Sec61β are shown as ER markers. (**e**) Accumulation of wt HA-LARP6 in microsomal fraction by rapamycin. Microsomal fraction and total cell lysate were analyzed as in (**d**) after treatment with rapamycin. (**f**) Microsomal accumulation of S348A/S409A mutant. Experiment as in (**e**), except S348A/S409A was analyzed. (**g**) Accumulation of wt HA-LARP6 in microsomal fraction after raptor knockdown. Microsomal fraction (MIC, left panel) and total cell lysate (TOT, right panel) of control cells (CON) and raptor knockdown cells (RPT) were analyzed by Western blot. The efficiency of raptor knockdown is shown in the bottom panel. (**h**) Microsomal accumulation of S348A/S409A in raptor knockdown cells. Experiment as in (**g**), except S348A/S409A was analyzed. (**i**) Interaction between LARP6 and Sec61. Microsomal fraction was prepared from cells expressing wt HA-LARP6 or S348A/S409A, immunoprecipitation (IP) was done using anti-Sec61β antibody (left panel, lanes 2 and 3) or control antibody (lane 1) and the IP material was analyzed using anti-HA antibody (MIC IP, left panel). Right panel, expression of the proteins in 10% of input.

The endogenous LARP6 is found in both the nucleus and cytosol^11^. We assessed if S348A/S409A and mTORC1 inhibition alters LARP6 distribution. Wt HA-LARP6 accumulated throughout the cytoplasm and nucleus, but after rapamycin or raptor knockdown it was depleted in the nucleus and found predominantly in the cytosol, with more intense staining in the perinuclear regions (Fig. 7a and b, upper panels). In contrast, S348A/S409A preferentially accumulated in the perinuclear region of the cytosol, while this distribution was not altered by rapamycin or by raptor knockdown (Fig. 7a and b, lower panels). These indicate that phosphorylation of S348/S409 is required for nuclear accumulation of LARP6.

Similarly, endogenous LARP6 was found in the cytoplasm and nucleus of control cells (Fig. 7c, left panels). However, LARP6 was found predominantly in the cytoplasm after rapamycin treatment or after raptor knockdown (Fig. 7c, right panels). Taken together, these results are indicative that mTORC1-dependent phosphorylation of S348/S409 contributes to LARP6 nuclear distribution.

The perinuclear localization of LARP6 is suggestive of increased association with the ER. We have reported that LARP6 targets collagen mRNAs to the ER membrane^8^. To investigate this possibility we isolated the microsomal fraction, which represents ribosome-enriched ER membranes, and compared the relative levels of S348A/S409A and wt HA-LARP6 in this fraction. Calnexin (CNX), a 67 kDa integral protein of ER membrane, which migrates as 90 kDa band in SDS-PAGE, was used as the ER marker^43^,^44^. The expression of both proteins in total cell lysate was similar, however, the relative abundance of S348A/S409A in the microsomal fraction was greater than that of wt LARP6 (Fig. 7d, top panel). This result is consistent with the notion that S348A/S409A localization around the perinuclear region is due to its accumulation at the ER membrane.

We also analyzed the microsomal distribution of wt HA-LARP6 after rapamycin treatment. Fig. 7e, top left panel, shows that in rapamycin treated cells twice as much of wt HA-LARP6 was found in microsome. The microsomal accumulation of S348A/S409A was high and was not further increased by rapamycin (Fig. 7f, top left panel). Similar results were obtained with raptor knockdown (Fig. 7g and h). Taken together, these results strongly suggest that phosphorylation of S348/S409 by mTORC1 is required for dissociation of LARP6 from the ER membrane and re-entry into the nucleus. Presence of raptor in microsome (Fig. 7g and h) has been indicated before^45^,^46^ and suggests that phosphorylation of LARP6 by mTOR can occur on the ER membrane.

Sec61 translocon is a protein-conducting channel composed of Sec61α/β/γ subunits that mediates co-translational insertion of most secretory and membrane proteins^47^,^48^,^49^. We have previously reported that LARP6 tethers collagen mRNAs to the ER membrane, where it interacts with Sec61^8^,^10^. Therefore, we assessed if the interaction of wt HA-LARP6 and S348A/S409A with Sec61 correlates with their accumulation in the microsomal fraction. Because greater amount of S348A/S409A accumulates in microsome, we adjusted the expression of wt HA-LARP6 and S348A/S409A so that similar amounts were found in the microsomal fraction (input in Fig. 7i). Under these conditions, more of S348A/S409A was pulled down with Sec61 than wt HA-LARP6, suggesting stronger interaction between the mutant and Sec61 (Fig. 7i, left panel, compare lanes 2 and 3). We concluded that LARP6 phosphorylation reduces its affinity for Sec61, allowing its release from the ER and shuttle into the nucleus.

## Discussion

LARP6 is a RNA binding protein which specifically binds collagen mRNA. The binding serves to target collagen mRNA to the ER membrane and tether RHA and STRAP^8^,^13^,^14^. These factors increase translational competitiveness of collagen mRNAs (RHA) and couple translation of collagen α1(I) and α2(I) mRNAs (STRAP). Coupled production of collagen polypeptides synchronizes the rate of modification to the rate of folding^8^,^12^,^14^,^50^. In contrast to the specific role of LARP6 in collagen mRNAs translation, mTOR pathway is involved in regulation of general translation by phosphorylating 4E-BP1 and S6K^22^,^24^. Our results are the first demonstration that mTOR signaling pathway regulates production of type I collagen via LARP6 phosphorylation, providing an example how translation of specific mRNAs is coupled to the general translation.

We recently identified eight phosphorylation sites on LARP6 and characterized the phosphorylation of S451 by AKT^31^. In this study we characterized the role of additional phosphorylations of LARP6. We show that (i) LARP6 is phosphorylated by mTORC1 on S348/S409, (ii) increased phosphorylation of LARP6 is observed during HSCs activation and is concomitant with increased collagen expression, (iii) S348A/S409A does not show the activation dependent phosphorylation in HSCs and has a dominant negative effect on collagen biosynthesis, (iv) the dominant negative effect is due to diminished rate of collagen secretion, (v) the interaction of S348A/S409A with STRAP, which couples translation of α1(I) and α2(I) mRNAs, is weakened, providing an explanation for its dominant negative effect, (vi) S348A/S409A predominantly accumulates at the ER membrane, while wt LARP6 shows such accumulation after mTORC1 inhibition, suggesting that phosphorylation of LARP6 is also required for its subcellular localization. Together, these results prompted us to postulate that LARP6 phosphorylation by mTORC1 has two functions. First, the phosphorylation increases the affinity of LARP6 for STRAP, coordinating the translation of collagen α1(I) and α2(I) mRNAs. Second, the phosphorylation reduces the accumulation of LARP6 at the ER membrane, allowing the protein to redistribute into the nucleus for new round of collagen mRNAs regulation. The results also provide novel rationale for the anti-fibrotic effect of rapamycin demonstrated in animal models of fibrosis.

Few reports implicated mTOR pathway in collagen biosynthesis. Leucine stimulated collagen α1(I) mRNA translation in HSCs via mTOR activation^51^. mTOR contributes to HSCs activation by regulating expression of TGF-β1 mRNA; TGF-β is the most potent profibrotic cytokine^27^,^52^. Our work shows that mTORC1 phosphorylates LARP6 at S348/S409 during HSCs activation and that these changes are important for upregulation of collagen. The first hint that LARP6 can be phosphorylated by mTOR was obtained when LARP6 phosphorylation sites were identified; five of the eight identified sites conformed to the mTOR consensus sequence^31^,^32^. This was further corroborated by altered electrophoretic mobility and pI of LARP6 after mTORC1 inhibition (Fig. 1). We have identified two mTORC1-dependent phosphorylation sites as the substitutions (S348A/S409A) which abolish rapamycin-induced pI change of the protein. Knockdown of raptor also could not change the phosphorylation status of S348A/S409A, while it had a profound effect on wt LARP6, further indicating that S348/S409 are the targets of mTORC1 (Fig. 3a,b and c). However, these results could not confirm that mTORC1 directly phosphorylates S348/S409 and we only concluded that mTORC1 is involved in LARP6 phosphorylation.

Type I collagen biosynthesis is unique among the synthesis of secretory proteins, because it involves coordinated translation, modifications and folding of collagen polypeptides into a triple helix^1^,^53^,^54^. Slow folding of collagen polypeptides results in hyper-modifications of the polypeptides and is seen in patients with osteogenesis imperfecta who have mutations affecting polypeptides folding^34^,^35^. S348A/S409A acts as a dominant negative protein in collagen biosynthesis by affecting excretion of collagen (Fig. 5). The slow rate of collage secretion by S348A/S409A is accompanied by hyper-modifications of collagen polypeptides, indicating their inefficient folding into the collagen trimer. Our previous work has shown that STRAP is essential for coordinating translation of collagen mRNAs and that it is tethered to collagen mRNAs by interaction with the CTER of LARP6^14^. STRAP knockout cells poorly secrete collagen and produce hyper-modified α2(I) polypeptides. The interaction between LARP6 and STRAP is less efficient when phosphorylation on S348/S409 is prevented either by inhibiting mTORC1 or by mutation into alanines (Fig. 6), and the cells poorly secrete collagen and produce hyper-modified collagen polypeptides. This suggests that mTORC1-dependent LARP6 phosphorylation is an essential mechanism which activates productive collagen synthesis.

mTORC1-mediated LARP6 phosphorylation increases its nuclear presence. S348A/S409A accumulates in increasing amounts at the ER membrane and interacts more strongly with Sec61 translocon. Similar effect is observed with wt LARP6 when mTORC1 is inhibited (Fig. 7). Based on our previous finding that LARP6 interacts with Sec61^8^,^10^, we postulate that in the absence of S348/S409 phosphorylation LARP6 has a diminished ability to dissociate from the ER membrane. This prevents its shuttling into the nucleus and participation in a new round of collagen mRNA metabolism^11^,^13^,^14^. Phosphorylation of LARP6 at the CTER does not change the binding affinity to collagen mRNAs, because this domain is dispensable for binding 5′SL. As the S348A/S409A also weakly interacts with STRAP, combination of these events results in dysregulated translation of collagen α1(I) and α2(I) mRNAs, the phenotype evident by hyper-modifications of polypeptides and inefficient secretion of type I collagen.

Substantial evidence has been presented that rapamycin exerts anti-fibrotic effect in fibrosis of multiple organs, including skin/lung/kidney/heart/liver^27^,^28^,^29^,^30^,^55^,^56^. Various mechanisms have been proposed for the anti-fibrotic effect; reduced infiltration of inflammatory cells and decreased expression of TGF-β1^29^, decreased platelet growth factor-induced proliferation of HSCs^27^, and destabilization of collagen mRNA^57^. This report gives another rationale for anti-fibrotic effects of rapamycin; inhibition of LARP6 phosphorylation by mTOR, resulting in inactivation of LARP6 in collagen biosynthesis.

In conclusion, we propose a mechanism of LARP6 activation in collagen biosynthesis. Involvement of mTORC1 in LARP6 phosphorylation at S348/S409 increases the affinity of LARP6 for STRAP. By recruiting STRAP, LARP6 couples translation of collagen α1(I) and α2(I) mRNAs, facilitating folding of the polypeptides into triple helix and rapid secretion. This phosphorylation also helps recycling of LARP6 from the ER membrane for another round of binding, partitioning and translation initiation of collagen mRNAs. The mTORC1 involvement of LARP6 phosphorylation takes place during the critical period of activation of HSCs, suggesting that it is one of the central events in fibrosis.

## Methods

### Plasmids and adenovirus construction

The CTER LARP6 was made as described previously^11^. Site directed mutagenesis of the single amino acids was done by QuickChange mutagenesis kit (Stratagene, 200523–5). The identity of all constructs was verified by sequencing.

Adenoviruses were generated by re-cloning of full-length LARP6 and mutants from pcDNA3 vectors into pAd-CMV-Track vector, followed by recombination in BJ5183 E.coli cells^58^. Adenoviruses were amplified in HEK293 cells and purified by cesium chloride density gradient centrifugation. All adenoviral vectors expressed the protein of interest and GFP, the later was encoded by an independent transcription unit^58^. GFP was used as a marker to estimate the efficiency of cell transduction.

### Chemicals and antibodies

Rapamycin was from Calbiochem (53123-88-9) and was prepared as 100 μM stock in DMSO. Torin 1 was purchased from Cell Signaling Technology (14379) and was dissolved as 100 μM stock in DMSO. Calf intestinal alkaline phosphatase (CIP) was from New England Biolabs (M0290S). Hexadimethrine bromide (Polybrene) was from Sigma-Aldrich (107689). Antibodies used were: anti-LARP6 antibody from Abnova (H00055323-B01P), anti-HA antibody from Sigma-Aldrich (H9658), anti-phospho AKT (S473), anti-pan AKT, anti-phospho S6K (T389), and anti-S6K antibodies from Cell Signaling Technology (12694, 4691, 9205, and 2708, respectively), anti-collagen α1(I) antibody from Rockland (600-401-103), anti-collagen α2(I) antibody from Santa Cruz Biotechnology (sc-8786), anti-calnexin antibody, human anti-fibronectin antibody and anti-STRAP antibody from BD Transduction Laboratories (610523, 610077, and 611346, respectively), rat anti-fibronectin from Millipore (AB1954), anti-β-actin antibody from Abnova (ab8227), anti-Sec61β antibody from Thermo Scientific (PA3-015) and anti-raptor antibody from Bethyl Laboratories (A300-553A-M).

### Cells and transfections

HEK293 cells, HEK293T cells (kind gift from R. Tomko) and HLFs were grown under standard conditions. HEK293 cells are human embryonic kidney cells^59^ and HEK293T cells are a variant of HEK293 cells that stably express SV40 large T antigen^60^. The expression of large T antigen allows replication of plasmids containing SV40 origin of replication and these cells are routinely used for packaging of lentiviruses. HEK293 cells are routinely used for protein expression studies and for packaging of adenoviruses. Transfections were done in 6-well plates with 1 μg of plasmid using TransIT-293 transfection reagent (Mirus, MIR2700). The cells were harvested 48 h after the transfections. Transduction of HLFs with adenoviruses was done at multiplicity of infection (MOI) of 500. With this MOI, between 95% and 100% of the cells were transduced. mTOR inhibitors, rapamycin (100 nM) or Torin 1 (100 nM) were added 0.5, 1, 2, and 24 h before collecting the cells. Cell extracts were made and analyzed by Western blot, 2DGE, or by immunoprecipitation.

### Production of lentivirus expressing raptor specific shRNA

Plasmid expressing raptor specific shRNA was made by cloning of double stranded oligonucleotide into the LKO.1 vector AgeI/EcoRI sites^61^. The sequence of the oligonucleotide was:

Raptor_shRNA sense: CCGGGGCTAG TCTGTTTCGA AATTTCTCGA GAAATTTCGA AACAGACTAG CCTTTTTG.

Raptor_shRNA antisense: AATTCAAAAA GGCTAGTCTG TTTCGAAATT TCTCGAGAAA TTTCGAAACA GACTAGCC.

Plasmid expressing raptor specific shRNA was co-transfected with pCMV-dR8.2 DVPR vector and pCMV-VSV-G vector at the ratio of 4:3:1 using LipoD293 Transfection Reagent (SignaGen Laboratories, SL100668) into HEK293T cells to allow packaging of the lentivirus. Virus containing supernatants were collected at 24 h intervals for three consecutive days and were used as source of virus. HLFs were plated in 6-well plates and the lentivirus transduction was carried out in media containing 8 μg/ml hexadimethrine bromide (polybrene). Transduction efficiency was determined by monitoring the viral marker, RFP. The knockdown of raptor was confirmed by Western blot.

### Rat hepatic stellate cell isolation and culture

Rat HSCs were isolated by perfusion of rat liver with 0.5 mg of pronase and 0.04 mg of collagenase per gram of animal weight, followed by centrifugation of the cell suspension over 20% Nykodenz gradient, as described^62^. All animal procedures were done according to the NIH guidelines and guidelines of Florida State University Animal Care and Use Committee (ACUC). The experimental protocol for isolation of HSCs was approved by the ACUC committee of Florida State University as the protocol #1428 on 7/30/14 and is valid for 3 years. The isolation of HSCs was performed strictly according to this protocol. After isolation, the cells were cultured in uncoated plastic dishes. HSCs Cell extracts were made at day 3 and day 5 and analyzed for AKT and mTOR expression by Western blot. For analysis of LARP6 phosphorylation, HSCs were transduced by adenovirus expressing wt LARP6 or mutants on day 2 or day 4 in culture and the cells and were harvested on day 3 or on day 5 for analysis. Treatment of HSCs with rapamycin (100 nM) was at day 5 for 2 h, followed by analysis of collagen levels by Western blot.

### Real-time PCR analysis

RNA was extracted from HLFs by using Trizol (Invitrogen). 1 μg of total RNA was used to prepare cDNA using the Superscript First Strand Synthesis System for RT-PCR (Invitrogen), according to the manufacturer’s instructions. 5 μl of 10-fold-diluted cDNA was used as in a SYBR Green qPCR assay (Applied Biosystems). The primers used for PCR amplification are shown in Table 1. Expression of collagen mRNAs was normalized to that of β-actin mRNA and statistical significance was determined using Student’s t test. P values of <0.05 were considered significant and the results are presented as means ± standard deviation (SD) (n = 3).

### Preparation of microsomal fraction

Microsomal fraction was prepared as reported before^63^, with minor modifications. HLFs were resuspended in 0.35 ml of hypotonic buffer and homogenized in Dounce Homogenizer. Diluted homogenate was layered on the top of 2.5 M sucrose and overlayed with 1.9 M and 1.3 M sucrose and centrifuged at 260,000 × g for 3 h at 4 °C. The band at the 1.3 M/1.9 M sucrose interface was recovered and centrifuged in a Beckman TLA120.2 rotor at 66,000 × g for 20 min at 4 °C. The pellet was lysed in 0.5% NP-40, 50 mM Tris-HCl, pH 7.5, 150 mM NaCl, 1 mM Dithiothreitol and protease inhibitors and used for Western blot or immunoprecipitations.

### Immunoprecipitations

HLFs were lysed in 50 mM Tris-HCl, pH 7.5, 150 mM NaCl, 0.5% NP-40, 1 mM Dithiothreitol, 170 μg/ml PMSF, 1 × protease inhibitors and cleared lysate was incubated with 1 μg of antibody for 3 h at 4 °C. 30 μl of equilibrated protein A/G-agarose beads was added, and incubation continued for 1 h. The beads were washed 3 times and analyzed by Western blot.

For CIP treatment, HA-LARP6 was overexpressed in HLFs, immunoprecipitated and treated with or without CIP at 37 °C for 3 h. The beads were then washed 4 times and analyzed by Western blot and 2DGE.

For immunoprecipitation experiments with microsomal fraction, 1 μg anti-Sec61β antibody was added and incubated for 30 min at 4 °C. 30 μl of equilibrated protein A/G beads were then added and incubation continued for an additional 30 min. The beads were washed 4 times and the samples analyzed by Western blot.

### Western blot

Cells were lysed in 50 mM Tris-HCl, pH7.5, 150 mM NaCl, 1% NP-40, 0.5% Sodium Deoxycholate, 0.1% SDS, 1 mM DTT, protease inhibitors and protein concentrations were estimated with the Bradford assay (Biorad, 500-0006), with bovine serum albumin (BSA) as standard. 40 μg of total cellular proteins was typically used for Western blot. For analysis of the medium proteins, equal numbers of cells were seeded and after 48 h the cells were washed 3 times with serum free medium. 600 μl of serum free medium was added per well and collagen accumulation was allowed to proceed for 1, 2, and 3 h. Serum free medium was collected and analyzed by Western blot.

For analysis of collagen protein stability in the medium samples, after collection, the serum free medium samples were incubated for additional 6 h at 37 °C.

### Two-dimensional gel electrophoresis

Cells were lysed in 50 mM Tris-HCl, pH 7.5, 150 mM NaCl, 0.5% NP-40 with protease inhibitors, and phosphatase inhibitors when necessary. The protein pellet or the immunoprecipitated HA-LARP6 was solubilized in 120 μl of rehydration buffer (7 M Urea, 2 M Thiourea, 2% CHAPS, 0.8% Ampholytes, 65 mM Dithiothreitol, and trace amount of Bromophenol blue) for 1 h at room temperature, and loaded onto Immobiline Dry Strip strips (7 cm, pH 3 to 10, GE Healthcare, 17-6001-11). An Ettan IPGphor 3 instrument (GE Healthcare) was used for the isoelectric focusing, according to the recommended protocol^64^. After focusing, the strips were equilibrated in Equilibration buffer A (0.375 M Tris-HCl, pH 8.8, 6 M Urea, 20% Glycerol, 2% SDS, 2% Dithiothreitol, bromophenol blue) for 10 min, followed by Equilibration buffer B (0.375 M Tris-HCl, pH 8.8, 6 M Urea, 20% Glycerol, 2% SDS, 2.5% Iodoacetamide, bromophenol blue) for 2 × 10 min. The second-dimension separation was done by laying strips onto 7.5% SDS PAGE, followed by Western blot. Immobilized strips showed slight batch to batch variations in the ampholyte distribution, so only the samples run on the same batch of strips were directly compared.

### Immunostaining of cells

HLFs were seeded onto glass coverslips. After treatment, cells were fixed in 4% paraformaldehyde in PBS for 10 min, followed by 3 washes with PBS. Cells were permeabilized with PBST (PBS containing 0.1% Triton X-100) for 10 min and blocked with PBTG (PBS containing 0.1% Triton X-100, 10% normal goat serum and 1% bovine serum albumin (BSA)) at room temperature for 2 h. Coverslips were incubated with primary antibody at 4 °C overnight. After 4 washes with PBS, secondary antibody diluted at 1:500 was added and incubated at room temperature for 1 h. Cells were washed 4 times with PBS and mounted with Prolong mounting solution containing DAPI. Images were taken by the EVOS FL Color fluorescence imaging system with 60 × objective.

## Additional Information

**How to cite this article:** Zhang, Y. and Stefanovic, B. mTORC1 phosphorylates LARP6 to stimulate type I collagen expression. *Sci. Rep.* **7**, 41173; doi: 10.1038/srep41173 (2017).

**Publisher's note:** Springer Nature remains neutral with regard to jurisdictional claims in published maps and institutional affiliations.

## Figures and Tables

**Table 1:** Real-time PCR primers used.

Gene names	Primer sequences
human collagen α1(I)	F: 5′-AGAGGCGAAGGCAACAGTCG-3′
	R: 5′-GCAGGGCCAATGTCTAGTCC-3′
human collagen α2(I)	F: 5′-CTTCGTGCCTAGCAACATGC-3′
	R: 5′-TCAACACCATCTCTGCCTCG-3′
human β-actin	F: 5′-GTGCGTGACATTAAGGAGAAG-3′
	R: 5′-GAAGGTAGTTTCGTGGATGCC-3′

## References

[b1] Kivirikko K. I. Collagen biosynthesis: a mini-review cluster. Matrix Biol. 16, 355–356 (1998). 9524355 10.1016/s0945-053x(98)90008-7

[b2] Smith K. & Rennie M. J. New approaches and recent results concerning human-tissue collagen synthesis. Curr. Opin. Clin. Nutr. Metab. Care 10, 582–590 (2007). 17693741 10.1097/MCO.0b013e328285d858

[b3] Rockey D. C., Bell P. D. & Hill J. A. Fibrosis — A Common Pathway to Organ Injury and Failure. N. Engl. J. Med. 372, 1138–1149 (2015). 25785971 10.1056/NEJMra1300575

[b4] Lindquist J. N., Marzluff W. F. & Stefanovic B. III. Posttranscriptional regulation of type I collagen. Am. J. Physiol. Gastrointest. Liver Physiol. 279, G471–G476 (2000). 10960344 10.1152/ajpgi.2000.279.3.G471

[b5] Lindquist J. N., Stefanovic B. & Brenner D. A. Regulation of collagen alpha1 (I) expression in hepatic stellate cells. J. Gastroenterol. 35, 80–83 (1999). 10779224

[b6] Stefanovic B. . Posttranscriptional regulation of collagen alpha1(I) mRNA in hepatic stellate cells. Mol. Cell. Biol. 17, 5201–5209 (1997). 9271398 10.1128/mcb.17.9.5201 PMC232371

[b7] Tsukada S., Parsons C. J. & Rippe R. A. Mechanisms of liver fibrosis. Clin. Chim. Acta 364, 33–60 (2006). 16139830 10.1016/j.cca.2005.06.014

[b8] Wang H. & Stefanovic B. Role of LARP6 and Nonmuscle Myosin in Partitioning of Collagen mRNAs to the ER Membrane. PLOS ONE 9, e108870 (2014). 25271881 10.1371/journal.pone.0108870 PMC4182744

[b9] Challa A. A. & Stefanovic B. A Novel Role of Vimentin Filaments: Binding and Stabilization of Collagen mRNAs. Mol. Cell. Biol. 31, 3773–3789 (2011). 21746880 10.1128/MCB.05263-11 PMC3165730

[b10] Stefanovic L., Longo L., Zhang Y. & Stefanovic B. Characterization of binding of LARP6 to the 5′stem-loop of collagen mRNAs: Implications for synthesis of type I collagen. RNA Biol. 11, 1386–1401 (2014). 25692237 10.1080/15476286.2014.996467 PMC4615758

[b11] Cai L., Fritz D., Stefanovic L. & Stefanovic B. Binding of LARP6 to the conserved 5′stem–loop regulates translation of mRNAs encoding type I collagen. J. Mol. Biol. 395, 309–326 (2010). 19917293 10.1016/j.jmb.2009.11.020 PMC2826804

[b12] Cai L., Fritz D., Stefanovic L. & Stefanovic B. Nonmuscle myosin-dependent synthesis of type I collagen. J. Mol. Biol. 401, 564–578 (2010). 20603131 10.1016/j.jmb.2010.06.057 PMC3674529

[b13] Manojlovic Z. & Stefanovic B. A novel role of RNA helicase A in regulation of translation of type I collagen mRNAs. RNA 18, 321–334 (2012). 22190748 10.1261/rna.030288.111 PMC3264918

[b14] Vukmirovic M., Manojlovic Z. & Stefanovic B. Serine-Threonine Kinase Receptor-Associated Protein (STRAP) Regulates Translation of Type I Collagen mRNAs. Mol. Cell. Biol. 33, 3893–3906 (2013). 23918805 10.1128/MCB.00195-13 PMC3811873

[b15] Hara K. . Raptor, a Binding Partner of Target of Rapamycin (TOR), Mediates TOR Action. Cell 110, 177–189 (2002). 12150926 10.1016/s0092-8674(02)00833-4

[b16] Kim D. . mTOR Interacts with Raptor to Form a Nutrient-Sensitive Complex that Signals to the Cell Growth Machinery. Cell 110, 163–175 (2002). 12150925 10.1016/s0092-8674(02)00808-5

[b17] Loewith R. . Two TOR Complexes, Only One of which Is Rapamycin Sensitive, Have Distinct Roles in Cell Growth Control. Mol. Cell 10, 457–468 (2002). 12408816 10.1016/s1097-2765(02)00636-6

[b18] Jacinto E. . Mammalian TOR complex 2 controls the actin cytoskeleton and is rapamycin insensitive. Nat. Cell Biol. 6, 1122–1128 (2004). 15467718 10.1038/ncb1183

[b19] Dos D. S. . Rictor, a Novel Binding Partner of mTOR, Defines a Rapamycin-Insensitive and Raptor-Independent Pathway that Regulates the Cytoskeleton. Curr. Biol. 14, 1296–1302 (2004). 15268862 10.1016/j.cub.2004.06.054

[b20] Hresko R. C. & Mueckler M. mTOR· RICTOR is the Ser473 kinase for Akt/protein kinase B in 3T3-L1 adipocytes. J. Biol. Chem. 280, 40406–40416 (2005). 16221682 10.1074/jbc.M508361200

[b21] Sarbassov D. D., Guertin D. A., Ali S. M. & Sabatini D. M. Phosphorylation and regulation of Akt/PKB by the rictor-mTOR complex. Science 307, 1098–1101 (2005). 15718470 10.1126/science.1106148

[b22] Burnett P. E., Barrow R. K., Cohen N. A., Snyder S. H. & Sabatini D. M. RAFT1 phosphorylation of the translational regulators p70 S6 kinase and 4E-BP1. Proc. Natl. Acad. Sci. USA 95, 1432–1437 (1998). 9465032 10.1073/pnas.95.4.1432 PMC19032

[b23] Brown E. J. . Control of p70 S6 kinase by kinase activity of FRAP *in vivo*. Nature 377, 441 (1995). 7566123 10.1038/377441a0

[b24] Hara K. . Regulation of eIF-4E BP1 phosphorylation by mTOR. J. Biol. Chem. 272, 26457–26463 (1997). 9334222 10.1074/jbc.272.42.26457

[b25] Martel R., Klicius J. & Galet S. Inhibition of the immune response by rapamycin, a new antifungal antibiotic. Can. J. Physiol. Pharmacol. 55, 48–51 (1977). 843990 10.1139/y77-007

[b26] Calne R. . Rapamycin for immunosuppression in organ allografting. Lancet 334, 227 (1989). 10.1016/s0140-6736(89)90417-0 2568561

[b27] Zhu J. . Rapamycin inhibits hepatic stellate cell proliferation *in vitro* and limits fibrogenesis in an *in vivo* model of liver fibrosis. Gastroenterology 117, 1198–1204 (1999). 10535884 10.1016/s0016-5085(99)70406-3

[b28] Bridle K. R. . Rapamycin inhibits hepatic fibrosis in rats by attenuating multiple profibrogenic pathways. Liver Transpl. 15, 1315–1324 (2009). 19790156 10.1002/lt.21804

[b29] Wu M. . Rapamycin attenuates unilateral ureteral obstruction-induced renal fibrosis. Kidney Int. 69, 2029–2036 (2006). 16732193 10.1038/sj.ki.5000161

[b30] Korfhagen T. R. . Rapamycin prevents transforming growth factor-α–induced pulmonary fibrosis. Am. J. Respir. Cell Mol. Biol. 41, 562–572 (2009). 19244201 10.1165/rcmb.2008-0377OC PMC2778163

[b31] Zhang Y. & Stefanovic B. Akt mediated phosphorylation of LARP6; critical step in biosynthesis of type I collagen. Sci. Rep. 6 (2016). 10.1038/srep22597 PMC4773855 26932461

[b32] Hsu P. P. . The mTOR-regulated phosphoproteome reveals a mechanism of mTORC1-mediated inhibition of growth factor signaling. Science 332, 1317–1322 (2011). 21659604 10.1126/science.1199498 PMC3177140

[b33] Thoreen C. C. . An ATP-competitive mammalian target of rapamycin inhibitor reveals rapamycin-resistant functions of mTORC1. J. Biol. Chem. 284, 8023–8032 (2009). 19150980 10.1074/jbc.M900301200 PMC2658096

[b34] Lamandé S. R. . Endoplasmic reticulum-mediated quality control of type I collagen production by cells from osteogenesis imperfecta patients with mutations in the proα1 (I) chain carboxyl-terminal propeptide which impair subunit assembly. J. Biol. Chem. 270, 8642–8649 (1995). 7721766 10.1074/jbc.270.15.8642

[b35] Tajima S., Takehana M. & Azuma N. Production of overmodified type I procollagen in a case of osteogenesis imperfecta. J. Dermatol. 21, 219–222 (1994). 8056893 10.1111/j.1346-8138.1994.tb01726.x

[b36] Bateman J. F., Mascara T., Chan D. & Cole W. G. Abnormal type I collagen metabolism by cultured fibroblasts in lethal perinatal osteogenesis imperfecta. Biochem. J 217, 103–115 (1984). 6421277 10.1042/bj2170103 PMC1153187

[b37] Bateman J. F., Mascara T., Chan D. & Cole W. G. A structural mutation of the collagen alpha 1 (I) CB7 peptide in lethal perinatal osteogenesis imperfecta. J. Biol. Chem. 262, 4445–4451 (1987). 3558348

[b38] Schalm S. S. & Blenis J. Identification of a conserved motif required for mTOR signaling. Curr. Biol. 12, 632–639 (2002). 11967149 10.1016/s0960-9822(02)00762-5

[b39] Schalm S. S., Fingar D. C., Sabatini D. M. & Blenis J. TOS motif-mediated raptor binding regulates 4E-BP1 multisite phosphorylation and function. Curr. Biol. 13, 797–806 (2003). 12747827 10.1016/s0960-9822(03)00329-4

[b40] Friedman S. Hepatic stellate cells. Prog. Liver Dis. 14, 101–130 (1995). 9055576

[b41] De Minicis S. . Gene expression profiles during hepatic stellate cell activation in culture and *in vivo*. Gastroenterology 132, 1937–1946 (2007). 17484886 10.1053/j.gastro.2007.02.033

[b42] Jiang F., Parsons C. J. & Stefanovic B. Gene expression profile of quiescent and activated rat hepatic stellate cells implicates Wnt signaling pathway in activation. J. Hepatol. 45, 401–409 (2006). 16780995 10.1016/j.jhep.2006.03.016

[b43] Bergeron J. J., Brenner M. B., Thomas D. Y. & Williams D. B. Calnexin: a membrane-bound chaperone of the endoplasmic reticulum. Trends Biochem. Sci. 19, 124–128 (1994). 8203019 10.1016/0968-0004(94)90205-4

[b44] Wada I. . SSR alpha and associated calnexin are major calcium binding proteins of the endoplasmic reticulum membrane. J. Biol. Chem. 266, 19599–19610 (1991). 1918067

[b45] Drenan R. M., Liu X., Bertram P. G. & Zheng X. F. S. FKBP12-Rapamycin-associated Protein or Mammalian Target of Rapamycin (FRAP/mTOR) Localization in the Endoplasmic Reticulum and the Golgi Apparatus. J. Biol. Chem. 279, 772–778 (2004). 14578359 10.1074/jbc.M305912200

[b46] Yadav R. B. . mTOR direct interactions with Rheb-GTPase and raptor: sub-cellular localization using fluorescence lifetime imaging. BMC Cell Biol. 14, 1–16 (2013). 23311891 10.1186/1471-2121-14-3 PMC3549280

[b47] Becker T. . Structure of monomeric yeast and mammalian Sec61 complexes interacting with the translating ribosome. Science 326, 1369–1373 (2009). 19933108 10.1126/science.1178535 PMC2920595

[b48] Dejgaard K. . Organization of the Sec61 translocon, studied by high resolution native electrophoresis. J. Proteome Res. 9, 1763–1771 (2010). 20112977 10.1021/pr900900x

[b49] Gogala M. . Structures of the Sec61 complex engaged in nascent peptide translocation or membrane insertion. Nature 506, 107–110 (2014). 24499919 10.1038/nature12950

[b50] Lamande S. R. & Bateman J. F. Procollagen folding and assembly: the role of endoplasmic reticulum enzymes and molecular chaperones. in Semin. Cell Dev. Biol. Vol. 10, 455–464 (Elsevier, 1999). 10597628 10.1006/scdb.1999.0317

[b51] de Obanos M. P. P., Zabalza M. J. L., Prieto J., Herraiz M. T. & Iraburu M. J. Leucine stimulates procollagen α1 (I) translation on hepatic stellate cells through ERK and PI3K/Akt/mTOR activation. J. Cell. Physiol. 209, 580–586 (2006). 16897753 10.1002/jcp.20790

[b52] Neef M., Ledermann M., Saegesser H., Schneider V. & Reichen J. Low-dose oral rapamycin treatment reduces fibrogenesis, improves liver function, and prolongs survival in rats with established liver cirrhosis. J. Hepatol. 45, 786–796 (2006). 17050028 10.1016/j.jhep.2006.07.030

[b53] Brass A., Kadler K., Thomas J., Grant M. & Boot-Handford R. The aromatic zipper: a model for the initial trimerization event in collagen folding. Biochem. Soc. Trans. 19, 365S (1991). 1794500 10.1042/bst019365s

[b54] Khoshnoodi J., Cartailler J.-P., Alvares K., Veis A. & Hudson B. G. Molecular recognition in the assembly of collagens: terminal noncollagenous domains are key recognition modules in the formation of triple helical protomers. J. Biol. Chem. 281, 38117–38121 (2006). 17082192 10.1074/jbc.R600025200

[b55] Yoshizaki A. . Treatment with rapamycin prevents fibrosis in tight‐skin and bleomycin‐induced mouse models of systemic sclerosis. Arthritis Rheum. 62, 2476–2487 (2010). 20506342 10.1002/art.27498

[b56] Yu S.-Y., Liu L., Li P. & Li J. Rapamycin inhibits the mTOR/p70S6K pathway and attenuates cardiac fibrosis in adriamycin-induced dilated cardiomyopathy. J. Thorac. Cardiovasc. Surg. 61, 223–228 (2013). 10.1055/s-0032-1311548 22684415

[b57] Poulalhon N. . Modulation of Collagen and MMP-1 Gene Expression in Fibroblasts by the Immunosuppressive Drug Rapamycin a Direct Role as an Antifibrotic Agent? J. Biol. Chem. 281, 33045–33052 (2006). 16914544 10.1074/jbc.M606366200

[b58] He T.-C. . A simplified system for generating recombinant adenoviruses. Proc. Natl. Acad. Sci. USA 95, 2509–2514 (1998). 9482916 10.1073/pnas.95.5.2509 PMC19394

[b59] Graham F., Smiley J., Russell W. & Nairn R. Characteristics of a human cell line transformed by DNA from human adenovirus type 5. J. Gen. Virol. 36, 59–72 (1977). 886304 10.1099/0022-1317-36-1-59

[b60] DuBridge R. B. . Analysis of mutation in human cells by using an Epstein-Barr virus shuttle system. Mol. Cell. Biol. 7, 379–387 (1987). 3031469 10.1128/mcb.7.1.379-387.1987 PMC365079

[b61] Stewart S. A. . Lentivirus-delivered stable gene silencing by RNAi in primary cells. RNA 9, 493–501 (2003). 12649500 10.1261/rna.2192803 PMC1370415

[b62] Weiskirchen R. & Gressner A. M. Isolation and culture of hepatic stellate cells. In Fibrosis Research 99–113 (Springer, 2005). 10.1385/1-59259-940-0:099 16118448

[b63] Stephens S. B., Dodd R. D., Lerner R. S., Pyhtila B. M. & Nicchitta C. V. Analysis of mRNA partitioning between the cytosol and endoplasmic reticulum compartments of mammalian cells. In Post-Transcriptional Gene Regulation 197–214 (Springer, 2008). 10.1007/978-1-59745-033-1_14 18369985

[b64] Boguth G., Harder A., Scheibe B., Wildgruber R. & Weiss W. The current state of two-dimensional electrophoresis with immobilized pH gradients. Electrophoresis 21, 1037–1053 (2000). 10786879 10.1002/(SICI)1522-2683(20000401)21:6<1037::AID-ELPS1037>3.0.CO;2-V

